# Improved mood despite worsening physical health in older adults: Findings from the International Mobility in Aging Study (IMIAS)

**DOI:** 10.1371/journal.pone.0214988

**Published:** 2019-04-08

**Authors:** Rebecca Lys, Emmanuelle Belanger, Susan P. Phillips

**Affiliations:** 1 School of Medicine, Queen’s University, Kingston, Ontario, Canada; 2 Center for Gerontology and Healthcare Research, Department of Health Services, Policy, and Practice, Brown University School of Public Health, Providence, Rhode Island, United States of America; 3 Family Medicine and Public Health Sciences, Queen’s University, Kingston, Ontario, Canada; Nathan S Kline Institute, UNITED STATES

## Abstract

**Objectives:**

Older adults experience increasing physical illness with age, but paradoxically, they frequently describe improvements in mood and self-rated health. The role of declining physical health as a risk for depression in elderly men and women remains unclear. We assessed whether declining physical health predicted changes in depression over time among seniors using data from the International Mobility in Aging Study (IMIAS).

**Methods:**

IMIAS is a longitudinal population-based study of older adults in Canada, Colombia, and Brazil. We assessed change in depression by comparing Center for Epidemiology–Depression (CES-D) scores for 1161 men and women between 2012 and 2016, and used multiple regression to identify whether changes in chronic health conditions, grip strength and self-rated health predicted change in depression over time.

**Results:**

Despite worsening physical health measured as chronic health conditions and grip strength, mean CES-D scores decreased from 8.15 (95% CI 7.70–8.60) in 2012 to 7.15 (95% CI 6.75 to 7.56) in 2016. Counterintuitively, women reported increased self-rated health despite having declining physical health, *p* = 0.004. Decreases in depressive symptoms were aligned with higher CES-D in 2012 and with increases in self-rated health among women and overall, and with high CES-D 2012 and increases in chronic health conditions in men, *p*s < 0.05.

**Conclusions:**

Mental health appears to be a fundamentally different construct than physical health in older adults, allowing seniors to experience improved mood despite declining physical health. Clinicians should not consider depression in elderly populations as an inevitability of aging.

## Introduction

Among those with depression, it is older adults who are most likely to attempt and complete suicide.[1] The role of concomitant physical disease, and the etiology of depression among seniors are poorly understood and confusing. Older adults are more likely than the young to have chronic illnesses such as cardiovascular disease, diabetes, or respiratory compromise, all recognized both as risk factors for and complications of depression.[2–5] Yet, paradoxically, most research demonstrates a decrease in prevalence of depression with increasing age.[6–8] There is evidence that some individuals, including seniors, with physical illness may experience a response shift of recalibrating their physical health expectations, reprioritizing their values, or reconceptualizing constructs in order to maintain their quality of life while experiencing a decline in health.[9–12]

The differential impact on women and men, that is, of sex/gender, adds to the confusion. In German and Canadian studies of older adults, increased chronic disease aligned with increased depressive symptoms in women.[13,14] However, a similar study in Italy found the reverse, with worsened physical health being more highly associated with depression in men.[15] Others have found that the association between chronic disease and depression diminishes with age but is unrelated to sex/gender.[6]

These conflicting findings are further muddied by variations in susceptibility to depression among older adults depending on country income and rural residency. An international cross-sectional study found that the prevalence of depression decreased with age in high-income, but not low- and middle-income countries.[6] Some have found that older adults living in metropolitan areas have decreased levels of depression relative to those in rural areas, whereas others find no association between rural setting and depression.[16,17] The role of setting is important in understanding the etiology of mood changes in seniors. If older adults in high-income and metropolitan settings, but not those in middle-income or rural settings, experience decreases in depression, this hints at the role of cultural or social contexts. Conversely, a decline in depression across all settings would suggest that seniors experience improved mood as they adapt to the aging process itself, regardless of other social factors.

Understanding how physical decline contributes to changes in mood will enable clinicians to better identify and treat older patients at risk. To our knowledge, no studies have previously used a longitudinal, population-based design to measure changes in mood and physical health across middle- and high-income countries. Our aim was to address this knowledge gap using data collected over four years from four study sites in Canada, Colombia, and Brazil to assess the relationships between physical decline and depression in older populations.

## Methods

### Data sources/study populations

The International Mobility in Aging Study (IMIAS), is a population-based longitudinal study of community-dwelling seniors, aged 65–74 on enrolment in 2012, and living in Kingston (Canada), St. Hyacinthe (Canada), Natal (Brazil), Manizales (Colombia), or Tirana (Albania). Each study setting reflects a different way of life, culture, and language, but inhabitants within each setting are relatively homogenous ethnically and culturally. Kingston is a university city in Ontario, St. Hyacinthe is an agricultural centre in Quebec, Manizales has a large coffee-growing region in the Andes, and Natal is a coastal city in a relatively low-income area of Brazil.[18] Because of uncertainty regarding the accuracy of the depression measure for Tirana, it was excluded from this study although preliminary analyses (not reported here) found that its inclusion did not change outcomes.

Respondents were recruited randomly from primary care medical centers in each setting. However, to satisfy local ethics committees' stipulations in Canada, researchers were not allowed to invite potential participants directly. Instead, patients of the target age in one large family practice received a letter signed by their primary care physician inviting those willing to participate to contact the study coordinator. In Manizales, the Public Health Insurance registry, which includes most adults age 65–74, was used to identify those to be invited to participate. In Natal, invitees were identified using neighbourhood primary care registers. Participation rates varied by study setting. In Kingston and St. Hyacinthe, 30% of those mailed the invitation contacted IMIAS, and 95% of that group chose to participate. Participation rates in Latin America were over 90%, which may reflect the appreciation respondents expressed at receiving free medical exams and blood tests. IMIAS recruitment and study design are described in greater detail in Zunzunegui et al., 2015.[18]

### Study design

**Fig 1:**
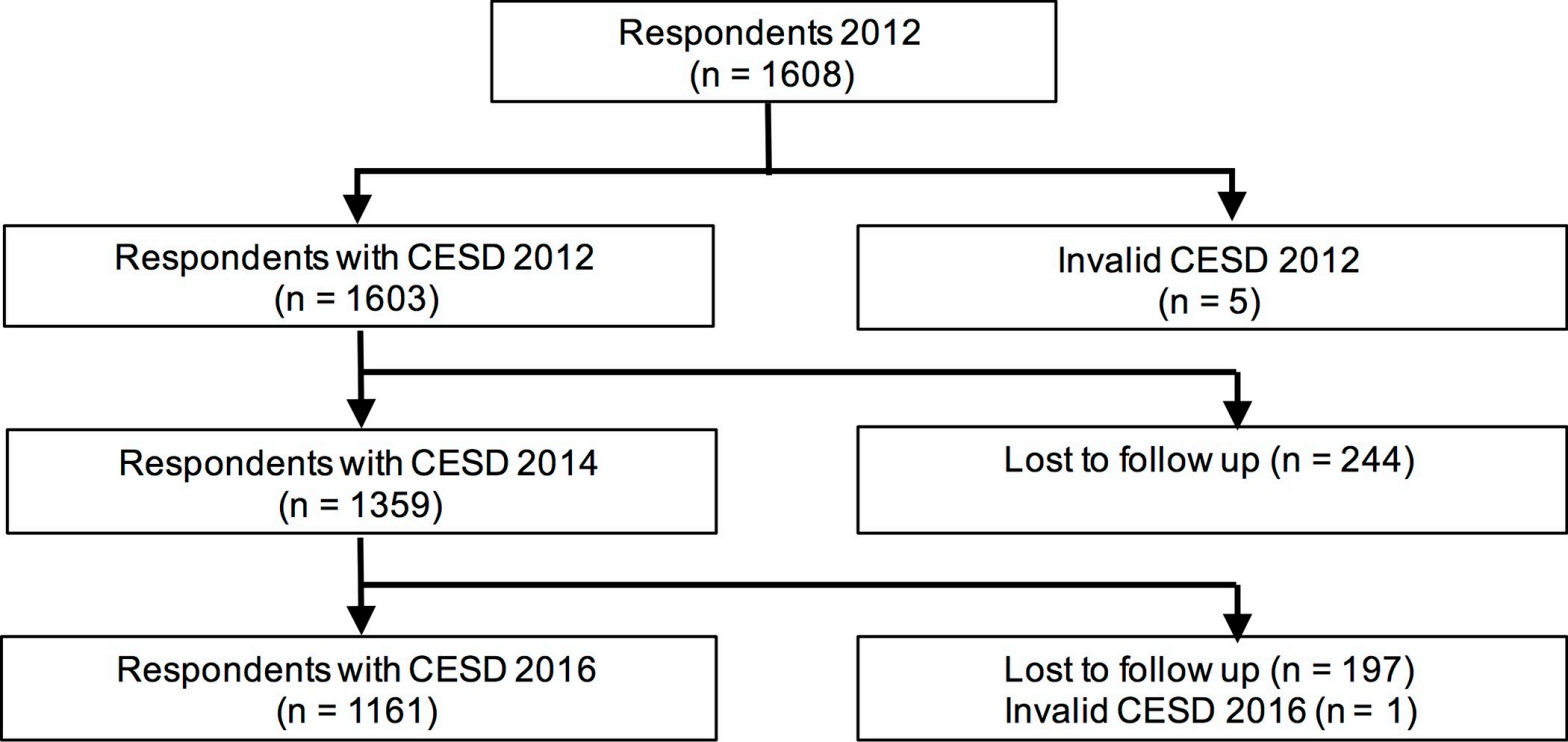
Flowchart illustrating respondent recruitment and withdrawal.

In 2012 approximately 200 community-dwelling men and 200 women were enrolled per site for a total of 768 men and 840 women (1608 total).[18] A small number of trained personnel conducted face-to-face interviews in offices (Kingston) or respondents’ homes (St. Hyacinthe, Manizales, Natal), and performed a battery of physical tests and measurements. Our analysis is limited to those who had a valid depression score at all three time points, as measured by the Center for Epidemiologic Studies—Depression (CES-D) in 2012, 2014, and 2016, resulting in 1161 respondents (Fig 1). Of the respondents who were lost to follow up, 123 moved, 85 died, 24 failed the cognitive screen, and 180 refused or were too sick to participate. Our inclusion criteria and the percentage of respondents who were excluded or lost to follow-up due to declining capacity or death are consistent with other longitudinal studies on aging including the Baltimore Longitudinal Study on Aging and the Italian Longitudinal Study on Aging.[19,20]. Earlier analyses showed that the proportion of all 2012 participants (ie including those subsequently lost to follow-up) with depression was 244/1601 or 15.2%[21]. This is nearly identical to the depression outcomes for 2012 of those who remained in the cohort, suggesting there was no substantive difference in mood at baseline across the two groups.

### Exclusion criteria

Those living in hospital or an assisted living facility were excluded from the initial cohort as our overall aim was to examine changes in mobility over time and therefore we enrolled participants who were able to live independently. Respondents remained enrolled in the study as long as they remained in the community, unless their cognitive ability declined such that they had four or more errors on the Leganes Cognitive Test (LCT).[18]

### Outcome measure: Change in depression

The primary outcome was change in depression (Δ depression), measured by the difference in respondents’ depression score in 2016 compared to 2012. We assessed depression using the Center for Epidemiological Studies Depression Scale (CES-D), a screen for depression which has been validated among older, community-dwelling adults and in English, French, Spanish, and Portuguese.[22–25] The CES-D consists of 20 questions about mood over the preceding 2 weeks, each assessed from 0 (rarely/never) to 3 (most/all of the time). Potential scores range of 0–60, and scores of 16 or more suggest a significant symptom burden indicative of depression.[26] Change in depression score was defined as the difference between CES-D 2016 and CES-D 2012, yielding a continuous variable, ranging from -40 (less depressed) to 32 (more depressed).

### Predictors of change in depression

The primary predictors examined were change in chronic health conditions (Δ CHC), change in grip strength (Δ grip) reflecting objective physical function, and change in self-rated health (Δ SRH), reflecting subjective perceptions of health.

CHC was assessed via a checklist of 8 self-reported diseases (hypertension, diabetes, malignancy, chronic lung disease, heart disease, cerebral aneurysm/stroke, osteoarthritis, and osteoporosis). We assessed baseline number of CHC in 2012 by asking respondents “Has a doctor or nurse ever told you that you have [condition]?” and in 2014 and 2016, we asked respondents, “Since we last saw you, has a doctor or nurse told you that you have [condition]?”. In 2014 and 2016, we used the total number of diseases endorsed at any point, accounting for duplication. CHC was a continuous variable with a possible range from 0–8, and an actual range of 0–7. Δ CHC was the increase in CHC from 2012 to 2016, with greater values indicating increases in CHC.

We measured hand grip strength with a grip dynamometer (Jamar Hydraulic Hand Dynamometer). The highest of three sequential measures at each time point with the dominant hand was utilized.[27] Grip strength is negatively correlated with all-cause mortality and poor health outcomes in healthy older adults, and thus reflects objective physical health.[28–30] We calculated Δ grip by comparing values for 2016 and 2012, with negative scores indicating decreases in strength on this continuous scale.

Self-rated health (SRH) was reported as 1 (very poor) to 5 (very good), assessed in 2012, 2014, and 2016. Poor self-rated health has been associated with increased depression in elderly populations, irrespective of objective physical health.[31,32] We calculated Δ SRH by comparing SRH scores in 2016 and 2012. Positive values reflected increased subjective physical health on this continuous scale.

### Covariates

Also included as potential explanations for change in depression were site, sex, age, income sufficiency, and smoking. Study site and sex were included because of discrepancies in prevalence of depression across countries,[6,16] and between women and men.[15] Smoking was included because of its association with depression and many of the comorbidities assessed within CHC.[33]

Income sufficiency was selected as the measure of socioeconomic status (SES) because it overcomes international discrepancies in income, cost of living, education, and standard of living across study sites. It has been used by others as an SES control variable in international studies of mental illness with diverse study sites.[34] Income sufficiency was defined as the extent to which income met the respondent’s needs from 1 (insufficient) to 3 (very sufficient).

### Statistical analysis

All analyses were performed using SPSS V. 24. Normality was assessed with visual inspection, Q-Q plots, box plots, and measuring skewness, and kurtosis. We used paired sample t-tests of changes in depression, CHC, grip strength, and self-reported health between 2012 to 2016 with a 5% threshold for statistical significance, two-tailed.[35] We reported on findings for the whole cohort and then for women and men separately. Data from 2014 are included to demonstrate time trends only.

Using multiple linear regression, we developed a model to predict Δ depression from 2012 to 2016 for men, women, and overall. Bivariate correlations identified those covariates significantly associated with Δ depression, with Spearman correlations being applied to analysis with one or more non-continuous variables, and Pearson correlations being used for the remainder. These, along with our primary predictor variables, were used as independent variables in Model 1. Only significant variables from Model 1 were then included in Model 2.

### Ethics statement

The study was approved by all relevant university or health sciences ethics committees: Queen’s University (Kingston), Centre de Recherche du Centre Hospitalier de l’Université de Montreal (St. Hyacinthe), the Universidad de Caldas (Colombia), and the Universidade Federal de Rio Grande do Norte (Brazil). Written informed consent was obtained from all participants at recruitment (2012).

## Results

Visual inspection with histograms, normal Q-Q plot, and box plots showed that the majority of data were approximately normally distributed. [36] Change in depression was within the normal range for males (skewness -0.260, SE 0.104, kurtosis 2.958, SE 0.209) and females (skewness -0.556, SE 0.099, kurtosis 2.689, SE 0.197).[37]

In 2012, the mean age of respondents was 68.97 and 52.9% were female. Income insufficiency varied by site, with Kingston and St. Hyacinthe having much lower rates of income insufficiency (2.8% and 6.1%) than Manizales and Natal (66.5% and 71.1%) (Table 1).

**Table 1:** Characteristics of respondents by study site.

	Total	Kingston, ON	St. Hyacinthe, QC	Manizales	Natal
Sex	*n* = 1161	*n* = 287	*n* = 294	*n* = 334	*n* = 246
Men	547 (47.1%)	127 (44.3%)	136 (46.3%)	161 (48.2%)	123 (50%)
Women	614 (52.9%)	160 (55.7%)	158 (53.7%)	173 (51.8%)	123 (50%)
Age 2012	*n* = 1161	*n* = 287	*n* = 294	*n* = 334	*n* = 246
65–69	674 (58.1%)	171 (59.6%)	188 (63.9%)	178 (53.3%)	137 (55.7%)
70–74	487 (41.9%)	116 (40.4%)	106 (36.1%)	156 (46.7%)	109 (44.3%)
Depression 2012	*n* = 1161	*n* = 287	*n* = 294	*n* = 334	*n* = 246
Not depressed	983 (84.7%)	261 (90.9%)	263 (89.5%)	262 (78.4%)	197 (80.1%)
Depressed	178 (15.3%)	26 (9.1%)	31 (10.5%)	72 (21.6%)	49 (19.9%)
Chronic health conditions 2012	*n* = 1147	*n* = 285	*n* = 283	*n* = 333	*n* = 246
0	203 (17.7%)	43 (15.1%)	49 (17.3%)	79 (23.7%)	32 (13.0%)
1	341 (29.7%)	79 (27.7%)	85 (30.0%)	106 (31.8%)	71 (28.9%)
2	321 (28.0%)	91 (31.9%)	75 (26.5%)	82 (24.6%)	73 (29.7%)
3	178 (15.5%)	45 (15.8%)	42 (14.8%)	47 (14.1%)	44 (17.9%)
4	75 (6.5%)	22 (7.7%)	19 (6.7%)	11 (3.3%)	23 (9.3%)
5	23 (2.0%)	5 (1.8%)	10 (3.5%)	5 (1.5%)	3 (1.2%)
6	5 (0.4%)	0 (0%)	3 (1.1%)	2 (0.6%)	0 (0%)
7	1 (0.1%)	0 (0%)	0 (0%)	1 (0.3%)	0 (0%)
Income sufficiency 2012	*n* = 1153	*n* = 287	*n* = 294	*n* = 326	*n* = 175
Insufficient (1)	423 (36.7%)	8 (2.8%)	18 (6.1%)	222 (66.5%)	175 (71.1%)
Sufficient (2)	382 (33.1%)	92 (32.1%)	145 (49.3%)	85 (25.4%)	60 (24.4%)
Very sufficient (3)	348 (30.2%)	187 (65.2%)	131 (44.6%)	19 (5.7%)	11 (4.5%)
Self-Rated Health 2012	*n* = 1159	*n = 286*	*n = 294*	*n = 334*	*n = 245*
Very poor (1)	9 (0.8%)	1 (0.3%)	0 (0%)	3 (0.9%)	5 (2.0%)
Poor (2)	44 (3.8%)	1 (0.3%)	4 (1.4%)	11 (3.3%)	28 (11.4%)
Fair (3)	350 (30.2%)	31 (10.8%)	39 (13.3%)	141 (42.2%)	139 (56.5%)
Good (4)	472 (40.7%)	122 (42.7%)	154 (52.4%)	132 (39.5%)	64 (26.0%)
Very good (5)	284 (24.5%)	131 (45.8%)	97 (33.0%)	47 (14.1%)	9 (3.7%)
Smoking 2012	*n* = 1160	*n* = 287	*n* = 294	*n* = 333	*n* = 246
Current non-smoker	1053 (90.8%)	274 (95.5%)	272 (92.5%)	289 (86.8%)	218 (88.6%)
Current smoker	107 (9.2%)	13 (4.5%)	22 (7.5%)	44 (13.2%)	28 (11.4%)

Across all sites, as respondents aged they reported fewer depressive symptoms with mean CES-D scores decreasing from 8.15 (95% CI 7.70–8.60) in 2012 to 7.15 (95% CI 6.75–7.56) in 2016 (p < 0.001). There were significant sex/gender differences identified. Women had higher CES-D scores at every stage (Table 2); however, both men and women experienced significant decreases in depression over time (*p*s < 0.001). Women also reported more CHC and had weaker grip strength, although men and women both underwent significant and similar worsening in CHC and grip strength (*p*s < 0.001). Nevertheless, women's SRH increased with aging (2012 = 3.81, 2016 = 3.90, *p* = 0.004), whereas men's did not (*p* > 0.05).

**Table 2:** Descriptive data: CES-D scores, chronic health conditions, grip strength, and self-rated health for men and women from 2012–2016.

	2012 Mean (95% CI)	2016 Mean (95% CI)	Paired samples *t* test (2012–2016) *t*, *p*
CES-D (out of 60)			
Overall (n = 1161)	8.15 (7.70–8.60)	7.15 (6.75–7.56)	*t* = 4.838, *p* < 0.001**+
Men (n = 545)	6.53 (6.00–7.07)	5.68 (5.18–6.18)	*t* = 3.266, *p* = 0.001**+
Women (n = 613)	9.59 (8.91–10.28)	8.46 (7.85–9.08)	*t* = 3.60, *p* < 0.001**+
Chronic health conditions (Out of 8)			
Overall (n = 1161)	1.72 (1.64–1.79)	2.08 (2.00–2.16)	*t* = -20.620, *p* < 0.001**+
Men (n = 545)	1.43 (1.32–1.53)	1.80 (1.69–1.90)	*t* = -14.294, *p* < 0.001**+
Women (n = 613)	1.97 (1.87–2.08)	2.34 (2.23–2.45)	*t* = -14.856, *p* < 0.001**+
Grip strength (kg)			
Overall (n = 1161)	28.83 (28.22–29.44)	26.98 (26.39–27.57)	*t* = 11.381, *p* < 0.001**+
Men (n = 545)	36.89 (36.13–37.65)	34.68 (33.93–35.44)	*t* = 7.559, *p* < 0.001**+
Women (n = 613)	21.64 (21.21–22.06)	20.08 (19.68–20.48)	*t* = 9.073, *p* < 0.001**+
SRH (out of 5)			
Overall (n = 1161)	3.84 (3.79–3.89)	3.91 (3.86–3.95)	*t* = -2.996, *p* = 0.003**+
Men (n = 545)	3.88 (3.82–3.95)	3.92 (3.86–3.99)	*t* = -1.209, *p* = 0.227
Women (n = 613)	3.81 (3.74–3.88)	3.90 (3.84–3.97)	*t* = -2.900, *p* = 0.004*+

Note: CI = confidence interval

* *p* < 0.05

** *p* < 0.01

+ Significant with Bonferroni adjusted p-value threshold of *p* < 0.00416.

**Fig 2:**
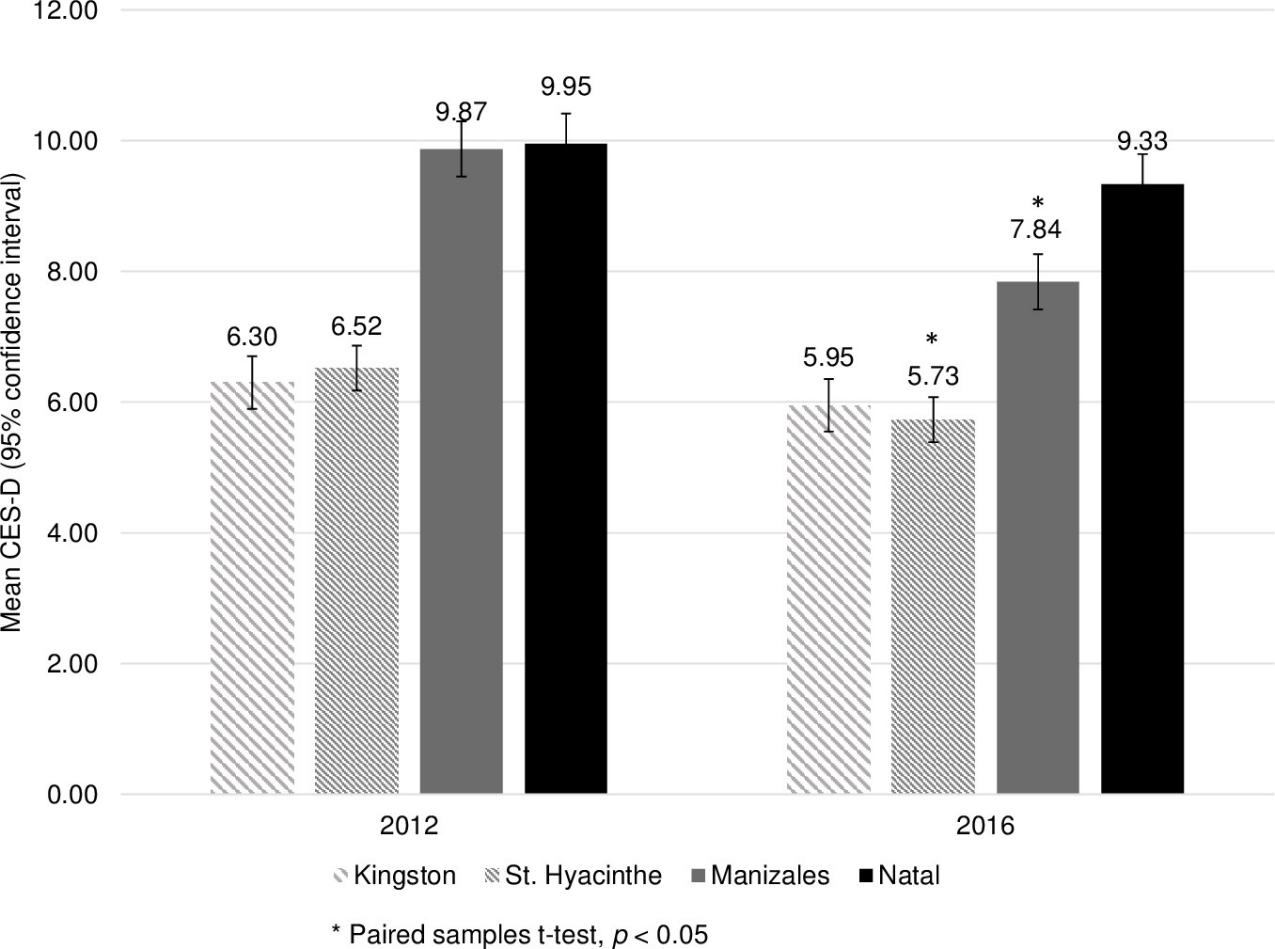
Mean depression scores over time.

CES-D scores in Latin America were higher (ie more depressed) than those in Canada at all stages. Overall CES-D scores decreased at all sites, although these site-specific findings were only significant for St. Hyacinthe and Manizales (*p*s < 0.05) (Fig 2).

In bivariate analyses, decreases in depression were significantly correlated with higher 2012 CES-D (i.e. greater depression initially) (*p* < 0.001). Respondents with insufficient income at baseline and those with increased SRH were also more likely to experience decreases in CES-D (*p*s < 0.001). Age, sex, and changes in objective physical health (i.e. Δ CHC, Δ grip, and smoking) were not significantly associated with Δ depression (*p*s > 0.05) (Table 3).

**Table 3:** Bivariate correlations of study covariates.

	Δ CES-D 2012–2016	CES-D 2012	Age 2012	Sex	Income Sufficiency 2012	Δ CHC	Δ Grip Strength	Δ SRH	Smoking 2012
Δ CES-D 2012–2016	1								
CES-D 2012	*r* = -0.548** p < 0.001	**1**							
Age 2012	*r* = -0.06 *p* = 0.847	*r* = 0.012 *p* = 0.679	1						
Sex^+^	^+^ *r* = -0.007 *p* = 0.817	^+^ *r* = 0.185** p **< 0.001**	^+^ *r* = 0.019 *p* = 0.508	1					
Income Sufficiency 2012	^+^ *r* = 0.099** p **= 0.001**	^+^ *r* = -0.301** p **< 0.001**	^+^ *r* = -0.030 *p* = 0.302	^+^ *r* = - 0.020 *p* = 0.503	1				
Δ CHC (2012–2016)	*r* = 0.024 *p* = 0.410	*r* = 0.007 p = 0.814	*r* = 0.027 P = 0.361	^+^ *r* = -0.002 *p* = 0.945	^+^ *r* = 0.041 *p* = 0.165	1			
Δ Grip Strength (2012–2016)	*r* = -0.058 *p* = 0.054	*r* = 0.060* p **= 0.045**	*r* = -0.054 *p* = 0.069	^+^ *r* = 0.045 *p* = 0.134	^+^ *r* = 0.091** p **= 0.002**	**r = -0.004** *p* **= 0.895**	1		
Δ SRH (2012 2016)	*r* = -0.158** p **< 0.001**	*r* = 0.109** p **< 0.001**	*r* = -0.038 *p* = 0.199	^+^ *r* = 0.033 *p* = 0.261	^+^ *r* **= 0.071** *p* **= 0.016**	*r* = -0.024 *p* = 0.413	*r* = 0.061* p **= 0.040**	1	
Smoking 2012	^+^ *r* = -0.046 *p* = 0.118	^+^ *r* = 0.099* p **= 0.001**	^+^ *r* = 0.001 *p* = 0.967	^+^ *r* = -0.075* p **= 0.010**	^+^ *r* = 0.090* p **= 0.002**	^+^ *r* = 0.010 *p* = 0.742	^+^ *r* = -0.015 *p* = 0.607	^+^ *r* = 0.021 *p* = 0.485	1

^+^ Spearman correlation

* p < 0.05

** p < 0.001

To refine understanding of predictors of Δ depression, we next developed a linear regression model using as independent variables those that were significantly correlated with Δ depression in bivariate analyses: CES-D 2012, Δ SRH, and income sufficiency (Model 1). Only CES-D 2012 was highly correlated with Δ depression, with Δ SRH and income sufficiency having a small correlation with Δ depression. We also included Δ CHC and Δ grip as objective measures of physical health as these were of primary interest despite their lack of significance in bivariate analyses. Data were analyzed for the whole sample, and then separately for men and women. Greater depression in 2012 remained highly predictive of a decrease in depression over time in men, women, and overall (*p*s < 0.001). Improved SRH also aligned with a decrease in depression overall and among women (*p*s < 0.001) but not in men. Increases in CHC were associated with increases in depressive symptoms for men (*p* = 0.007) but not for women or among the cohort, overall (*p*s > 0.05). With the inclusion of multiple predictors, income insufficiency ceased to be significant and Δ grip remained insignificant (*p*s > 0.05). In our final model (Model 2), greater baseline CES-D and increases in SRH were associated with decreases in depression overall and in women, R^2^ = 0.310 and R^2^ = 0.325, respectively, whereas for men, higher 2012 CES-D scores and absence of increased chronic disease were the significant predictors, R^2^ = 0.308 (Table 4).

**Table 4:** Multiple Linear Regression–Predicting Δ Depression from 2012 to 2016.

Variable	Unstandardized Coefficient	Standard Error	*p*-value
All			
	Model 1		
Constant	3.737	0.554	< 0.001**
CES-D 2012	-0.502	0.023	< 0.001**
Δ SRH	-0.920	0.234	< 0.001**
Δ CHC	0.305	0.288	0.290
Δ grip (kg)	-0.023	0.033	0.480
Income sufficiency	-0.432	0.223	0.052
	Model 2		
Constant	2.977	0.248	< 0.001**
CES-D 2012	-0.481	0.022	< 0.001**
Δ SRH	-0.925	0.229	< 0.001**
Men			
	Model 1		
Constant	3.175	0.693	< 0.001**
CES-D 2012	-0.560	0.036	< 0.001**
Δ SRH	-0.605	0.310	0.051
Δ CHC	0.948	0.365	0.010*
Δ grip (kg)	-0.025	0.034	0.466
Income sufficiency	-0.444	0.276	0.109
	Model 2		
Constant	2.221	0.337	< 0.001**
CES-D 2012	-0.526	0.034	< 0.001**
Δ CHC	0.999	0.368	0.007*
Women			
	Model 1		
Constant	4.642	0.847	< 0.001**
CES-D 2012	-0.502	0.032	< 0.001**
ΔSRH	-1.214	0.338	< 0.001**
Δ CHC	-0.265	0.432	0.539
Δ grip (kg)	-0.039	0.064	0.540
Income sufficiency	-0.455	0.340	0.182
	Model 2		
Constant	3.649	0.385	< 0.001**
CES-D 2012	-0.489	0.030	< 0.001**
ΔSRH	-1.104	0.328	0.001**

* p < 0.05

** p < 0.001

## Discussion

As respondents aged, they reported fewer depressive symptoms despite declining physical health. This improvement in mood was consistent for men and women, and across high- and middle-income settings. The most consistent predictor of a decrease in depression was a higher initial CES-D. This could demonstrate nothing more than regression to the mean, however the consistency of the finding across settings and groups suggests something more. While objectively-measured physical health declined and number of chronic diseases increased, SRH increased or remained stable, implying that subjective assessments of one's health sometimes extend beyond a count of chronic diagnoses to include elements of mental well-being.

Sex/gender differences emerged when men and women were examined separately. Improved SRH was associated with decreased depression among women, despite women having poorer objective physical health, overall. It is possible, therefore, that what we measure when we measure SRH may be gendered. Women, in particular, may take an inclusive view of subjective health, incorporating self-rated mental wellness along with their physical health. In men, increase in CHCs aligned with decreases in mood, which supports literature findings that depression in men is associated with mobility and functional impairment.[15] In keeping with others, we found that women reported more depressive symptoms than men at any given time, but improvements in mood with aging were similar for all.[6,15,16] Our findings suggest that mental health is fundamentally different from and unrelated to physical health in older adults although among women, SRH may include both.

Our research is consistent with existing evidence in showing decreased depression and increased SRH over time among older adults, despite declines in objective physical health.[31,32] However, unlike others who found a decline in depression in high-income and metropolitan, but not middle-income or rural settings, we found that depressive symptoms decreased in all settings, although middle-income sites had higher CES-D scores at any given time.[6,16] Prior research was cross-sectional and may not have fully accounted for higher baseline rates of depression in middle-income countries. Surprisingly, while IMIAS respondents with insufficient income reported more depressive symptoms at baseline, they experienced greater improvements in mood over time than their economically better off counterparts. Our study’s longitudinal and international design provides new insight into the role of context and income sufficiency on mood. Because men and women across settings experienced fewer depressive symptoms over time, our results support the concept that older adults experience a fundamental shift in outlook as part of their aging experience despite increasing physical illness or socioeconomic position.

Socioemotional selectivity theory (SST) postulates that the elderly prune memories to favour positive recollections, and this may explain why aging brings improved mood.[7,38] According to SST, older adults may screen out negative thoughts (e.g. about declining physical health) in favour of positive ones (improved mood and self-rated health).[7] Others speculate that elders may develop adaptive coping techniques including response shifts in order to maintain their well-being while facing physical, economic, and social stressors.[9,10,12] This may help explain why those with insufficient income reported more depressive symptoms at baseline, but experienced greater improvements in mood with time. Our findings reinforce the concept that older adults shift their conceptualization of health risks, detaching these from a sense of successful aging and, in the process, experiencing an improvement in mood. Those with higher baseline depression scores seemed to experience the greatest shift, as they had larger decreases in depression over time.

There is clinical importance to these findings. Physicians should not dismiss increasing depression in the elderly as an inevitable outcome of declining physical health. Rather, doctors should recognize that lower mood in older adults is a red flag for a serious yet treatable condition. In addition, physicians might put less emphasis on enumerating risks and numbers of chronic diseases, and instead help older patients build a sense of successful aging despite declining objective physical health.

Our longitudinal study design with an international sample allows us to shed light on the pre-existing, conflicting research findings about depression and physical illness in older adults across different settings. While factors such as sex/gender, chronic illness, income insufficiency can contribute to a higher baseline prevalence of depression, older adults across diverse settings appear to have decreases in depressive symptoms as they age.

### Limitations

Our study results must be interpreted within limitations: only community-dwelling respondents who passed a cognitive screening test were included. Older adults with dementia, and those in assisted living facilities have higher levels of depression, but as fewer than 1% of those 65–74 live in collective dwellings, their exclusion is unlikely to have dramatically altered outcomes.[8,39] Our research is limited to those who participated in IMIAS across the four years of data collection and does not account for those who died or withdrew from IMIAS. We do not attempt to generalize to those with dementia or to those with the most severe illnesses. We did not control for medication use, and it is possible that antidepressants started between 2012 and 2016 may have affected associations between mood, CHC, and SRH, particularly for those with a higher burden of depressive symptoms at baseline. We used a single, albeit evidence-based indicator of physical function, that is, grip strength. The variation across sites in depression and other variables examined almost certainly speaks to beneficial or harmful social norms and values in each setting. However, we could not measure these and therefore can only speculate as to what they might be. Finally, IMIAS enrolled those aged 65–74 in 2012, and we cannot confidently generalize findings to the oldest old.

## Conclusion

Our longitudinal, international study demonstrates that decreased mood is not an inevitable consequence of physical illness in older adults. While risk factors such as sex/gender, chronic illness, and income insufficiency can contribute to a higher baseline prevalence of depressive symptoms, adults across middle and high income settings appear to have improvements in mood as they age. Health care providers treating older adults should not dismiss depression as an expected consequence of aging. Instead they might help patients develop a sense of successful aging despite increasing physical comorbidities.
